# 

**Published:** 1891-07

**Authors:** 


### Society Meetings.

The American Association of Obstetricians and Gynecologists will hold its fourth annual meeting, at the New York Academy of Medicine, 17 West Forty-third street, in the City of New York, Thursday, Friday, and Saturday, September, 17, 18, and 19, 1891, under the presidency of Dr. Adam H. Wright, of Toronto. All physicians interested in the discussion of subjects pertaining to Abdominal Surgery, Obstetrics, and Gynecology are invited to attend without further formal notice. By order of the Executive Council.

WILLIAM WARREN POTTER, M. D.,
*Secretary.*

---

The Mississippi Valley Medical Association will hold its seventeenth annual session at St. Louis, Wednesday, Thursday, and Friday, October 14, 15, and 16, 1891. A large attendance, a valuable programme, and a good time are expected. The members of the medical profession are respectfully invited to attend. C. H. Hughes, M. D., President, 500 North Jefferson avenue, St. Louis. E. S. McKee, M. D., Secretary, 57 West Seventh street, Cincinnati. I. N. Love, M. D., Chairman of the Committee of Arrangements, 301 Grand avenue, St. Louis.

The Annual Meeting of the Medical Society of Chautauqua will be held in Mayville on the second Thursday (9th) of July, 1891, when a number of interesting papers will be read.

---

### Book Reviews.

### BOOKS AND PAMPHLETS RECEIVED.

Plain Talks on Electricity and Batteries, with Therapeutic Index. For General Practitioners and Students of Medicine. By Horatio R. Bigelow, M. D., Permanent Member of the American Medical Association; Fellow of the British Gynecological Association; Member of the Philadelphia Electro-Therapeutic Society, etc. 12mo, pp. vi.—85. Philadelphia: P. Blakiston, Son & Company. 1891.

Text-Book of Medical Jurisprudence and Toxicology. By John J. Reese, M. D., Professor of Medical Jurisprudence and Toxicology in the University of Pennsylvania; Late President of the Medical Jurisprudence Society of Pennsylvania; Honorary Member of the New York Academy of Anthropology; Corresponding Member of the New York Medico-Legal Society, etc. Third edition, revised and enlarged. Small octavo, pp. xvi.—666. Philadelphia: P. Blakiston, Son & Company. 1891.

Practical Points in the Management of Some of the Diseases of Children. By I. N. Love, M. D., Professor of Diseases of Children, Clinical Medicine and Hygiene in the Marion-Sims College of Medicine, St. Louis; Consulting Physician to the City Hospital, St. Louis, etc., etc. The Physicians' Leisure Library, Detroit: George S. Davis. 1891. Price, cloth, 50 cents; paper, 25 cents.

The Action, Therapeutic Value, and Use of the Carlsbad Sprudel Salt (Powder Form), and its Relation to the Carlsbad Thermal Water. By Dr. W. Jaworski, Demonstrator at the University of Krakow. Clinical experimental researches made at the University Clinic of Prof. Korczynski, in Krakow, with a Dietary by the Translator, A. L. A. Toboldt, M. D., Assistant Demonstrator of Pharmacy, University of Pennsylvania; Editor *Journal of Balneology and Medical Clippings*. 8vo, pp. viii.—100. Philadelphia: P. Blakiston, Son & Company. 1891.

Massage. A Primer for Nurses. By Sarah E. Post, M. D., Lecturer before the Training Schools for Nurses, connected with Bellevue, Mt. Sinai, St. Luke's, and Charity Hospitals, New York. Second edition, with seven original photographs. 12mo, pp. 47. New York: The Nightingale Publishing Company. 1891.

Report of Twenty-four Abdominal Operations in the Philadelphia Hospital. By E. E. Montgomery, M. D. Reprint: The Philadelphia Hospital Reports, Vol. I. 1890.

Hodgkin's and Allied Diseases. By W. H. Bergtold, M. D. Reprint: *Buffalo Medical and Surgical Journal*, April, 1891.

The Aseptic Method as Applied to Intra-Nasal Surgery. By John O. Roe, M. D., Rochester, N. Y., Fellow of the American Laryngological Association, etc. Reprint: *The Medical News*, March 28, 1891.

Stricture of the Oesophagus from Interstitial Thickening of its Walls. A Fibroid Hypertrophy. By John O. Roe, M. D., Rochester, N. Y. Reprint: *The New York Medical Journal*, March 14, 1891.

Aspiratore – iniettatore. Del Dr. Emilio Pittarelli. Reprint: Proceedings Italian Society of Internal Medicine. 1890.

Proceedings and Addresses at a Sanitary Convention, held at Alpena, Mich., July 10 and 11, 1890, under the direction of a Committee of the State Board of Health and a Committee of the Citizens of Alpena. Supplement to the Report of the Michigan State Board of Health for the Year 1890.

A Consideration of Some of the Parts of a Microscope Stand, of Interest to Pharmacists. By Dr. H. M. Whelpley, F. R. M. S. Reprint: *Popular Science News.*

The Causation of Influenza, Sometimes Called by its French Name, "La Grippe." By Henry B. Baker, M. D., Secretary Michigan State Board of Health. Reprint.

Abstract of the Proceedings of the Michigan State Board of Health. Annual Meeting, April 14, 1891. Reported by the Secretary of the Board.

Consideration of the Legal Status of Midwifery in the State of New York. By F. Park Lewis, M. D., Buffalo, N. Y. Reprint: *The North American Journal of Homeopathy*, April, 1891.

Ueber die Endergebnisse der Vaginalen Total Exstirpation wegen Carcinom an der Königlichen Frauenklinik zu Dresden. Von Eduard Leisse, Erstem Assistenten. Sonderabdruck a. d. *Archiv f. Gynäkologie*, Bd. XL., Heft 2.

Ventrofixatio uteri und Schwangerschaft. Von G. Leopold. Sonderabdruck aus dem *Centralblatt für Gynäkologie*, 1891. No. 16.

Practical Use of the Metric System. By A. L. Benedict, M. D., Buffalo, N. Y., Attending Physician Fitch Provident Dispensary. Reprint: *Medical and Surgical Reporter*, March 7, 1891.

Operative Procedures in Acute General Suppurative Peritonitis. By W. E. B. Davis, M. D., Birmingham, Ala. Read by invitation before the Medical Society of the State of New York, at Albany, February 3, 1891.

Report of the Health Officer of the Port of New York to the Commissioners of Quarantine of the State of New York. 1891. By William M. Smith, M. D., Health Officer.

A New Operation for Prolapsus of the Anterior Vaginal Wall. By Andrew F. Currier, M. D., New York. Read before the Section of Obstetrics and Diseases of Women and Children, at the Meeting of the American Association, Nashville, Tenn., May, 1890. Reprint: *Annals of Gynecology and Pediatry.*

Treatment of the Opium-Neurosis. By Stephen Lett, M. D., Medical Superintendent of the Homewood Retreat, Guelph, Ont. Report of a paper read before the American Medical Association at Washington, May, 1891.

Sixth Annual Report of the Board of Health of the City of Newport, R. I. For the year 1890.

Resection of the Optic Nerve. By L. Webster Fox, M. D. Second paper. Reprint: *The Medical and Surgical Reporter*, May 30, 1891.

Sixth Annual Announcement and Catalogue of the Western Pennsylvania Medical College, Pittsburgh. Sessions of 1891–1892.

### INDEX.

Page.

ABDOMEN, Penetrating Gun-Shot Wounds of . . . . . . . . . 28
Abscess, Evacuation of Psoas, or Lumbar through Vagina . . . 419
of the Liver, Novel Treatment of . . . . . . . . . 421
Acardia, Edematous . . . . . . . . . 527
Adenoid Growths in Naso-Pharynx . 95
Aid Association, N. Y. Physicians . . 753
Anastomosis, Intestinal. . . . . . 614
Anemia, Pernicious . . . . . . 229, 357
Anemic Murmurs, Mechanism of . . 262
Anesthesia, Local . . . . . . . . . . 292
in Small Surgical Operations . . . . . . . . 93
Antipyrin in Diseases of Children . . 96
Ankle-Joint Disease . . . . . . . . 281
Aphasia, Unique Case of . . . . . . 31
Apoplexy, First Therapeutic Measures in . . . . . . . . . . . . . . . 362
Aristol. . . . . . . . . . . . . . . 363
Arthritis, following Purulent Conjunctivitis . . . . . . . . . . . 485
Asthenopia, Muscular . . . . . . . 683
Atelectasis Pulmonum . . . . . . . 65
Aulde, John, M. D. Notes on Administration of Iron . . . . . . . 492

BACTERIA, Poisonous Products of intestinal . . . . 228
and their Relations to Disease . . . . . 711
Bacterial Poisons . . . . . . . . . 678
Bad Breath . . . . . . . . . . . . . 99
Beef Juice, Wyeth's . . . . . . . . . 128
Benedict, A. L., M. D. Muscles of the Forearm . . . . . . 211
Fistula of the Parotid Gland. 423
Menthol in Local Treatment of Erysipelas . . . . . . 606
Bennett, Alice, M. D. Insanity as a Symptom of Bright's Disease . . . 129
Bergtold, William H., M. D. Hodgkin's and Allied Diseases. . . . . 544
Bibliographie, Médicale Pharmaceutic, et Vétérinaire, Revue, de . . . 63
Blephorospasm, Treatment of . . . . 682
Blood, Alkalinity of . . . . . . . . . 676
Books and Pamphlets Received . 59, 120, 251, 315, 380, 444, 509, 634, 703, 771
Brain Grafting, Successful . . . . . 231
Brains, Examination of, in the Feeble Minded. . . . . . . . . . . . . 488
Bright's Disease, Insanity as a Symptom of. . . . . . . . . . . . . 129

Page.

Buswell, Henry C., M. D. Statistical Report of 300 Consecutive Cases of Labor. . . . . . . . . . . . . 12

CAPUT COLI, Anatomical Position of . . . . . . . . . . . 577
Carbonic Anhydride, Influence of Work in Exhalation of . . . . . . 676
Castration, Double, for Tuberculous Orchitis . . . . . . . . . . . . 479
Cataract, Treatment of Immature. . . 485
Cerebral Localization . . . . . . . 29
Cesarean Section, Indications for . . 471
Chloroform, Danger of Adminstering . . . . . . . . . . . . . . . 750
Cholera. Is it a Neurosis? . . . . 608
Club-Foot . . . . . . . . . . . . . 725
Conjunctivitis, Prevention of Purulent, in Infants . . . . . 406
Note on Subjective . . 267
Conjunctiva, Boracic Acid in Diseases of. . . . . . . . . . . . . . . 293
Court Reception at Berlin. . . . . . 179
Coxitis, Pathology and Treatment of Later Stages of . . . . . . . . . 385
Cranium, Temporary Resection of . . 612

DAGENAIS, A., M. D. Varicose Veins . . . . . . . . . . . 600
Deafness, Caused by Fracture and Concussion of the Temporal Bone. 1
Diathesis, Rheumatic and Gouty, in Diseases of Throat . . . . . . . 427
Dictionary, a New Medical . . . . . 58
Diseases of Children, Cyclopedia of . 58
Dorr, Samuel G., M. D. Puerperal Fever . . . . . . . . . . . . . . 164

EFFUSIONS into the Thorax . . 7
Electricity, Observations on Therapeutic Value of . . . 257
Epiglottis, New Method of Raising . 428
Epilepsy, Treatment of . . . . . . 609
Epithelioma, Cure of Sebaceous, by Resorcin . . . . . . . . . . . . 288
Equino-Varus, Double Congenital . . 532
Ergot in Facial Neuralgia . . . . . 99
Erie County Eye, Ear, and Throat Infirmary. . . . . . . . . . . . . 63
Erysipelas, New Methods of Treatment in . . . . . . . 234
Menthol in Treatment of . 606

Page.
Errata . . . . . . . . . . . . . . . 437
Evans, Chas. S., M. D., Bacteria and their Relations to Disease . . . . 711
Eye, Foreign Bodies in. . . . . . . 486
and Cardio-Vascular System, Points of Connection Between. 484
-Strain, Recognition of . . . . . 78
Examination, Civil Service, for Physicians . . . . . . . . . . . 448, 629

FACIAL NEURALGIA, Ergot in. 99
Falk, Jacob M., M. D., Prevention of Purulent Conjunctivitis in Infants . . . . . . 406
Fat, Absorption of. . . . . . . . . 356
Fetal Head, New Method of Delivering . . . . . . . . . . . . . 214
Fistula, Recto-Vaginal, Operative Treatment of . . . . . . . . . . 641
Food Supply . . . . . . . . . . . 173
Foot, Fibro-Sarcoma of. . . . . . . 667
Forearm, Muscles of . . . . . . . . 211
Freckles, Removal of . . . . . . . 273

GLOBULIN, a Bacteria-Killing . 678
Glucose, Destruction of, in Blood and Chyle . . . . . . . . . . . . . . 357
Gluten as a Food . . . . . . . . . 615
Goitre, Græfe's Lid-Sign in Exophthalmic . . . . . . . . . . . . . 294
Goldberg, Jacob, M. D. Note on Subjective Conjunctivitis . . . . . . . 267
Grove, B. H., M. D. Hematoma Auris . . . . . . . . . . . . . . 205
Gum-Lancing in Children . . . . . 364
Gynecology, Minor, and Major Pelvic Troubles. . . . . . . . . . . . . 193

HAYD, H. E., M. D. Letters from Paris. . . . . 37–103
Replies to J. P. and J. H. 236
Hallucinations, Census of. . . . . . 184
Hay-Fever. Its Etiology, Pathology, and Treatment . . . . . . . . . 85
Heart, Condition of, in Anemia . . . 355
Heat, The, and its Effects . . . . . 47
Health, How to Preserve . . . . . 59
Bulletins . . . . . . . 62, 123
256, 318, 382, 447, 511, 639, 702
Hematoma Auris . . . . . . . . . 205
Hemoglobin, Is Free, Present in Blood-Plasma of Splenic Vein. . . . . . 228
Hemorrhoids, Treatment of, by Excision . . . . . . . . . . . . . 268
Hernia, Infantile . . . . . . . . . 726
Hinkle, F. W., M. D. Hay-Fever . 85
Hip-Joint Disease After Typhoid Fever. . . . . 280
Hip-Joint Disease, Adjusted Locomotion in Treatment of . . . 668
Fixation in Treatment of . . . . 728
Hodgkin's Disease . . . . . . . . . 544
Hospital Graduate Degree . . . . . 240
Hoffman, Joseph, M. D. Surgical Treatment of Effusions into the Thorax . . . . . . . 7
Joseph, M. D. Perforation of Uterus with Curette Operation, Recovery . . . 22
Hot Water, Therapeutics of. . . . . 591
Humerus, Open Arthrotomy in Subclavicular Dislocation of . . . . . . 705
Hubbell, A. A., M. D. New Test for Determining Equilibrium of Ocular Muscles . . . . . . . . . 19
Hypnotism in Relation to Surgery . . 488
Hypnotic Anesthesia, Amputation Under. . . . . . . . . . . . . . 611
Hypnotized, Ophthalmoscopic Examination of the Eyes of the . . . . 233
Hysterectomy, Discussion of Vaginal, with Cases . . . . . . . . . . . 396

INSANITY, as a Symptom of, Bright's Disease . . 129
Rythmic Sound in Treatment of. . . . 611
Infants, Act for Prevention of Blindness in . . . . . . . . . 245
Food. . . . . . . . . . . 344
Feeding of, DeLancey Rochester, M. D. . . . . . . . 75
Feeding, Milk Sugar in . . . 98
Knee- and Ankle-Joints, Disease in . . . . . . . . . . 721
Infectious Diseases, Danger Limit to . 175
Fundamental Factors in the Causation of . . . . 230
Insane, Recent Legislation for . . . 610
Iron, Notes on Administration of . . 492

JACKSON, EDWARD, M. D. Recognition of Eye-Strain by the General Practitioner . . . . . . . 78

KAUROSIS VULVÆ. . . . . . 160
Knee-Joint, Excision of . . . 664
Koch's Tuberculosis Cure . . . . . 345
Lymph, Notes on Use of . . 582
Krauss, William C., M. D. Muscular Atrophies . . . . . . . . . . . 513
Kyphosis . . . . . . . . . . . . . 731

Page.

LABOR, Statistical and Clinical Report of 300 Consecutive Cases of . . . . . . . . . . . . . 12
Larynx, Diagnosis and Therapeutics of Cancer of . . . . . . . 425
Intubation in Specific Stenosis of . . . . . . . . . . . . . 710
Lead Poisoning by Carbonated Beverages . . . . . . . . . . . . . . . 225
Legislation, Important Medical . . . 44
Library, State Medical. . . . . . . . 567
License and Registration of Physicians, New Law Affecting . . . . . . . 46
Literary Notes . . . . . . . . . . . 242
Liver, Normal Storage of Iron in . . 358

MAJOR PELVIC TROUBLES Traceable to Minor Gynecology. . . . . . . . . . . . . . 193
Manley, Thomas H., M. D., Anatomical Position of the Caput Coli . . . 577
Mattison, J. B., M. D., Renal Status of Opium Habitués . . . . . 332
Maxilla, Resection of Inferior. . . . 481
McPherson, George W., M. D., Presidential Address Erie Co. Medical Society. . . . . . . . . . . . . . 460
Medical Law, Another New . . . . 244
Legislation, Important . . . 44
College Announcements . . . . . . . . . . . 62, 123, 319
Medicine, A Year's Progress in . . . 587
Membrana Tympani, Ruptures of . . 94
Micro-Organisms, Peptic and Diastatic Ferments of . . . . . . . . . . 225
Millions in It, There's . . . . . . . 127
Minor Gynecology and Major Pelvic Troubles . . . . . . . . . . . . . 193
Muscular Atrophies . . . . . . . . . 513
Mütter Lectures . . . . . . . . 429, 751
Mynter, Herman, M. D., Pathology and Treatment of Later Stages of Coxitis. . . . . 385
Surgical Cases at Sisters' of Charity Hospital . . . . . 478
Myopia, Removal of Lens in . . . . 487

NASAL DISEASE and Neurasthenia . . . . . . . 337
Erectile Tissue, Anatomy of . . . . . . . . . . 426
Cavity, Development of, After Birth . . . . . 427
Nerve Grafting . . . . . . . . . . 610
Neuromimesis . . . . . . . . . . . . 725
Neuralgia, A Treatise on . . . . . . 59
Neurasthenia and Nasal Disease . . 337

OCULAR MUSCLES, New Test for Determining Equilibrium of. 19

Page.

Obstetric Points, Some . . . . . . . 298
Opium Habitués, Renal Status of . . 332
Osteo-Malacia, Case of. . . . . . . . 280
Osteotomy, Double for Bow Legs . . 483
Double-Supra Condyloyd of Femur . . . . . . . . . 727
Ovarian and Ligamentous Cysts Coexisting in Same Patient . . . . 457

PATENTS, Recent, in Medicine and Surgery . . . . . . . 124, 512
Paralysis, Pressure . . . . . . . . . 608
Park, Roswell, M. D., Tetanus and Tetany . . . . . . 350
In Honor of . . . . . 692
Parmenter, John, M. D., Open Orthrotomy in Subclavicular Dislocation of the Humeris . . . . . . . 705
Parotid Gland, Fistula of . . . . . 423
Penrose, Charles B., M. D. Treatment of Hemorrhoids by Excision . . . 268
Peritonitis, Cases of Tubercular, Treated by Abdominal Section . . 321
Pilocarpine in Ocular Therapeutics . 293
Dryness of the Tongue in . . . . . . . . . 363
Pituitary Body, Calcareous Degeneration of . . . . . . . . . . . . . 286
Poliomyelitis, Anterior . . . . . . . 723
Plasters, Caoutchouc . . . . . . . . 33
Porro-Cesarean Section, A Successful. 40
Post-Graduate, A String of Pearls from the . . . . . . . . . . . . . 627
Potter, F. H., M. D., Treatment of Hay-Fever. . . . . . . . 88
F. H., M. D., Neurasthenia and Nasal Disease . . . . 337
Frank H., M. D., Croupous Rhinitis . . . . . . . . . 525
William Warren, M. D., Ovarian and Ligamentous Cysts in same Patient . . . . . 457
William Warren, M. D., State Medical Examining and Licensing Board . . . . . . 529
Pott's Disease, Importance of Thorough Examination in Suspected. 537
Lamnectomy for . . . . 665
Price, Joseph, M. D., Major Pelvic Troubles and Minor Gynecology . . 193
Prize, The Alvarenga . . . . . . . 320
The Mattison . . . . . . . . 447
Proteids of Liver and Kidney Cells . 228
Protoplasm, Influence of Electricity on. 232
Ptomaines, Recent Researches on . . 32
in Urine in Mumps . . . 225
Puerperal Fever. . . . . . . . . . . 164
Report of Cases of . 274
Pulmonary Phthisis, Gastro-Intestinal Disorders, and Diet in. . . . . . 356

Page.
RADIUS, Non-Union of Fractured. 279
Recto-Vaginal Fistula, Operative Treatment of . . . . . 641
Rectum, Scirrhus of, and Excision . 482
Reed, Charles A. L., M. D., Discussion of Vaginal Hysterectomy, with Cases . . . . . . . . . . . . 396
Registration and License of Physicians, New Law Affecting . . . . 46
Restraint, Mechanical, in Hospitals for the Insane . . . . . . . . . 449
Rhinitis, Croupous . . . . . . . . 525
Atrophic. . . . . . . 95, 426
Ricketts, Edwin, M. D., Exploratory Incision as an Aid to Diagnosis of Abdominal Disease . . . . . . 224
Rochester, De Lancey, M. D., Substitute for Milk in Infant Feeding . . . . . . 75
Mechanism of Anemic Murmurs. . . . . . . 262
Roe, John O., M. D., Intubation in Specific Stenosis of Larynx . . . . 718
Rohé, George H., M. D., Some Obstetric Points . . . . . . . . . . 298
Rolando, New Method of Determining Position of Fissure of . . . . 613
Ross, James F. W., M. D., Description of Edematous Acardia. 527
Tubercular Peritonitis Operated by Abdominal Section. 321

SALICYLBROMANILID . . . . 747
Salol in Acute Tonsillitis . . . 96
Sänger, Dr. Max, Operative Treatment of Recto-Vaginal Fistula . . . . . 641
Scotoma, Central . . . . . . . . . 680
Shimwell, Benj. S., M. D., New Method of Delivering the Fetal Head . . . . . . . . . . . . . . 214
Smith, Charles N., M. D., Kaurosis Vulvæ . . . . . . . . . . . . 160
Smith, Eugene A., M. D., Report of Cases of Puerperal Fever . . . . . 274
Spectacle Frames, Construction and Adaptation of . . . . . . . . . 592
Spine, Lateral Curvature of . . . . . 667
Spina Bifida . . . . . . . . . . . . 480
Snow, Irving M., M. D., Atelectasis Pulmonum . . . . . . . . . . . 65
Somnal, Notes on . . . . . . . . . 359
Sound, Rythmic, in Treatment of Insanity . . . . . . . . . . . . 543
State Medical Examining and Licensing Board . . . . . . . . . 529
Medical Examining Boards. National Conference of . . . . . . . 627
Sternum, Excision of, for Melanosarcom . . . . . . . . . . . . . . . 478
Street Cleaning in Large Cities . . . 569

Page.
Surgical Treatment of Effusions into the Thorax . . . . . . 7
Surgical Cases at Sisters' of Charity Hospital . . . . . . . . . 478
Synechiæ, Division of Anterior . . . 682

TEMPORAL BONE, Fracture and Concussion of, as a Cause of Deafness . . . . . . . . . . . . 1
Tendon-Reflex, Absence of Patellar in Keratitis . . . . . . . . . . . . 683
Tetanus and Tetany . . . . . . . . 350
Therapeutic Notes . . . 35, 99, 362, 489
Thorner, Dr. E., Notes on Koch's Lymph . . . . . . . . . . . . . 582
Thomas, Charles Hermon, M. D., Construction of Spectacle Frames . 592
Thorax, Surgical Treatment of Effusions into the . . . . . 7
Apparatus for Measuring . . 418
Trachoma, Treatment of, with Bichloride of Mercury . . . . . . . . . 294
Transfusion of Mixtures of Blood and Salt Solution . . . . . . . . . 677
Tuberculosis, Laparatomy in Peritoneal . . . . . . . . . . . . . . 292
Turnbull, Lawrence, M. D., Fracture and Concussion of the Temporal Bone (Contre-Coup) as a Cause of Deafness . . . . . . . . . . . . 1

UNIVERSITY, State of New York, Examinations Department . . . . . . . . . . . 366, 756
Urethral Discharges, Contagiousness of . . . . . . . . . . . . . . . 684
Urethral Injections . . . . . . . . 290
Urine, Albumin in, Containing Bacteria . . . . . . . . . . . . . . 677
Uterus, Perforation of, with Curette . 22

VARICOSE VEINS . . . . . . 600
Volume XXX.—Alpha . . . . 43
—Omega . . . 754
Volkmann, Richard Von, Memorial to . . . . . . . . . . . . . . . 64

WARTS, Iodine in the Treatment of . . . . . . . . . . 235
Wende, Ernest, M. D., Observations on Therapeutic Value of Electricity . . . . . . . 257
Ernest, M. D., The Polyscope . . . . . . . . . . 42
Grover Wm., M. D., On Plasters Containing Caoutchouc . . . . . . . 33
Wise, P. M. M. D., A Year's Progress in Medicine . . . . . . . . 587

Page.
Woodbury, Frank, M. D., Notes on Somnal . . . . . . . . . . . . . 359
Wounds and Ulcers, Dry Treatment of Open . . . . . . . . . . . . 291

YEAST, Action of, on Animal and Human Organism . . . . . . 677

### Obituary:

Barker, Fordyce, M. D. . . . . . . . 761
Baxter, Jedediah H., M. D., Surgeon-General U. S. Army . . . . . . . 371
Bradnack, Fowler, M. D. . . . . . . 694
Burwell, George N., M. D. . . . . . . 693
Carpenter, Arthur B., M. D. . . . . . 303
Fox, Sidney Allan, M. D. . . . . . . 372
Helwig, A. F., M. D. . . . . . . . . 695
Leidy, Joseph, M. D. . . . . . . . . 761
Levis, Richard J., M. D. . . . . . . . 302
McReynolds, William H., M. D. . . . 372
Parkes, Charles T., M. D. . . . . . . 631
Sprague, William B., M. D. . . . . . 570
Stewart, James L., M. D. . . . . . . . 372
Van Pelt, William, M. D. . . . . . . 248
Vaughn, Frank O., M. D. . . . . . . 570

### Medical College Notes:

Chicago Polyclinic . . . . . . . . . . 113
Niagara University, Medical Department . . . . . . . . . . . . 182, 759
Kentucky School of Medicine . . . . 759
University of Buffalo, Medical Department . . . . . . . . . 183, 568, 695

### Hospitals and Infirmaries:

Buffalo General Hospital . . . . . . . 302
Nurses Training School . 762
State Hospital . . . . . . . 761
Woman's Hospital . . . . . 692
Erie County Eye, Ear, and Throat Infirmary . . . . . . . . . . . . . 368
Johns Hopkins Hospital, Volume Descriptive of . . . . . . . . . . . . 127

### Societies:

American Association of Obstetricians and Gynecologists . . . . 112, 186, 220, 384, 512, 762
Medical Association . . . 502
Addresses before . 757
Society of Microscopists . . 50
Public Health Association . . . . . . . . . . . . 51, 414
Electro-Therapeutic Association . . . . . . . . 501
Gynecological Society . 51, 184
Association of Railway Surgeons, The National . . . . . . . . . . 502, 698
Baltimore Gynecological and Obstetrical Society . . . . . . . . . . . . 471
Buffalo Medical and Surgical Association . 17, 85, 164, 247, 421, 745
Microscopical Club . . . . . 247
Obstetrical Society . . . . . . . . .247, 274, 303, 502, 600
Pathological Society . . . . . . . . . . . . . 247, 350, 540
Medical Union . . . . . . . . 373
Canadian Medical Association . . . 52
District Medical Society, Hudson, N. J. . . . . . . . . . . . . . . . 369
Kentucky State Medical Society . . . 763
Medical Association of Central and Western New York . . . . . . . . . 631, 696, 763
Society of Chautauqua Co. . . . . . . . . . . . . . 752, 764
Society of the County of Erie . . . . . 459, 698, 732
Society of the County of Monroe . . . . . . . . . 762
Society of the State of New York . . . . . 247, 373, 437
Society of the State of Ohio. 763
Mississippi Valley Medical Association . . . . . . . . . . 112, 245, 762
New York State Medical Association, Fourth District Branch . 674
Academy of Medicine . . 301
Section on Orthopedic Surgery, 279, 418, 532, 664, 720
Pan-American Medical Congress . . 630
Philadelphia Obs etrical Society . . . 22
County Medical Society. . . . . . . 78, 214, 268, 592
Southern Surgical and Gynecological Association . . . . . . . . . . 246, 300
Seventh International Congress of Hygiene and Demography . . . . 372
Virginia Medical Society. . . . . . . 224

### Correspondence:

Hayd, H. E., M. D., Letters from Paris . . . . . . . . . 37, 103
H. E., M. D., Replies to J. P. and J. H. . . . . . . . . 236
Letter from J. P. and J. H., in Reply to Dr. Hayd . . . . . . . . . . . 167
King, Clarence, M. D., The Hospital Graduate Degree . . . . . . . . . 240
Marcy, Henry O., M. D. . . . . . . . 295
Williams, Herbert U., M. D. . . 429, 751
Pohlman, Julius, M. D. . . . . 620, 752

Page.

### Personals:

Abbott, Mrs. Lyman . . . . . . . . 50
Brush, Edward N., M. D. . . . . . 558
Conkling, Wm. J., M. D. . . . . . 49
Culbertson, J. C., M. D. . . . . . . 760
Dagenais, Alphonse, M. D. . . . . . 186
Dorr, L. Bradley, M. D. . . . . . . 370
Foster, Frank P., M. D. . . . . . . 571
Folwell, M. B., M. D., . . . . . . . 693
Frederick, C. C., M. D. . . . . . . 630
Grant, Henry Y , M. D. . . . . . . 187
Hamilton, John B., M. D. . . . . . 758
Krauss, William C., M. D. . . 186, 441
Lee, Benjamin, M. D. . . . . . . . 502
Lewis, Theo. G., D. D. S. . . . . . 758
Low, Frank W., D. D. S. . . . . . 441
Lomax, William, M. D. . . . . . . 112
Lydston, James A., M. D. . . . . . 248
McAllister, Eleanor, M. D. . . . . . 186
McCray, G. W., M. D. . . . . . . 693
McCullough, Mr. George B. . . . . 48
McMurtry, Lewis S., M. D. . . 501, 630
Mynter, Dr. and Mrs. Herman . . . 758
O'Brien, E. C. W., M. D. . . . . . 693
Park, Roswell, M. D. . . . . . . . 558
Pohl, Gustav A., M. D. . . . . . 370
Powell, Seneca D., M. D. . . . . . 302
Price, Joseph, M. D. . . . . . . 49, 569
Ross, T. Haven, M. D. . . . . . . 370
Reed, Charles A. L., M. D. . . . . 501
Senn, Nicholas, M. D. . . . . . . 630
Sexton, John C., M. D. . . . . . . 370
Starr, Elmer E., M. D. . . . . . . 49
Stockton, Charles G., M. D. . . . 187
Stoddart Brothers. . . . . . . . . 48
Storck, Edward, M. D. . . . . . . . 247
Stone, Isaac S., M. D. . . . . . . 302
Sutherland, Charles, M. D., Surgeon General U. S. Army . . . . . . . 370
Van Klein, Carl H. . . . . . . . . 758
Walker, H. O., M. D. . . . . . . 693
Ward, W. M., M. D. . . . . . . . 758
Wetmore, George T., M. D. . . . . 50
Weidner, Carl, M. D. . . . . . . . . 370
Wyman, Walter, M. D. . . . . . . 759

### Reviews:

Anderson: Mineral Springs and Health Resorts of California . . . 187
Ballou: Equine Anatomy and Physiology . . . . . . . . . . . . . 575
Barkan: How to Preserve Health . 59
Billings: Index Catalogue . . . . 379
Birnbaum: Koch's Method in Tuberculosis . . . . . . . . . . . 634
Brennan: Patient's Record . . . . 379
Browne: Koch's Remedy in Throat Consumption . . . . . . . . . . 699
Capp: The Daughter . . . . . . . 700
Clark: Physical Diagnosis, etc. . . 507
Corning: Treatise on Headache, Neuralgia, etc. . . . . . . . . . 505
DaCosta: Medical Diagnosis . . . 375
Davis: Pharmacology of Newer Materia Medica . . . . . . . . . 190
Dock: Text-Book of Materia Medica for Nurses . . . . . . . . . . 702
Dowse: Lectures on Massage and Electricity . . . . . . . . . . . 312
Edinger: Structure of Central Nervous System . . . . . . . . . . 632
Elmer: Physicians' Hand-Book . . 444
Foster: Encyclopedic Medical Dictionary. . . . . . . . . . . . . 504
Fowler: Dictionary of Practical Medicine. . . . . . . . . . . . 632
Gerard: Treatment of Sterility in Both Sexes . . . . . . . . . . 443
Gould: A New Medical Dictionary . 58
Gross: Treatise on Impotence and Sterility in the Male . . . . . . 314
Gruber: Text-Book on Diseases of Ear . . . . . . . . . . . . . . 571
Hamilton: Fractures and Dislocations . . . . . . . . . 767
Treatment of Headaches. 508
Hare: Text Book of Practical Therapeutics . . . . . . . . . 188
Epilepsy . . . . . . . . . 374
Horwitz: Compend of Surgery . . 314
Hurd: A Treatise on Neuralgia . . 59
Jackson: Essentials of Refraction and Diseases of the Eye . . . . . 191
Keating: How to Examine for Life Insurance . . . . . . 119
Cyclopedia of Diseases of Children . . . . . . 58
Pocket Medical Lexicon . 702
Keen: Operation Blanks . . . . . 507
Kelsey: Diseases of Rectum and Anus . . . . . . . . . . . . . . 113
King: Stories of a Country Doctor . 117
Lea: Year Book of Treatment . . . 633
Loomis: Text - Book of Practical Medicine. . . . . . . . . . . . 52
McDowell, Ephraim, Biography of . . 315
Mack: Philosophy in Homeopathy . 192
Martin: Essentials of Minor Surgery . . . . . . . . . . 576
Essentials of Surgery . . . 700
Massey: Electricity in Diseases of Women . . . . . . . . . . . . 118
May: Diseases of Women . . . . 118
Maxwell: Terminologia Polyglotta . 119
Mills: Text-Book of Comparative Physiology . . . . . . . . . . . 503
Morris: Essentials of Practice of Medicine . . . . . . . 310
Compend of Gynecology . . 701

Page.
Nancrede: Essentials of Anatomy . 250
Owen: Post-Mortems . . . . . . . . 508
Parvin: Science and Art of Obstetrics . . . . . . . . . . . . . . . 309
Paschkis: Cosmetics . . . . . . . 770
Piffard: Diseases of the Skin . . . 766
Powell: Essentials of Diseases of Children . . . . . . . . . . . . . 377
Potter: Compend of Human Anatomy . . . . . . . . . . . . . . . 576
Prudden: Dust and its Dangers . . 633
Story of the Bacteria . . 633
Purdy: Diabetes . . . . . . . . . . 770
Roberts: Manual of Modern Surgery. . . . . . . . . . . . . . . 441
Rowell: Book for Advertisers . . . 768
Sajous: Annual of the Universal Medical Sciences . . . . . . . . . 116
Satterlee: Rheumatism and Gout . . 114
Salmonsen: Bacteriological Technology . . . . . . . . . . . . 379
Shoemaker: Ointments and Oleates. 506
Society Transactions. State: New York, North Carolina, 55
Maryland . . . . . . 55, 508
Mississippi, Missouri . . . 55
Association of Obstetricians and Gynecologists . . . . 764
American Gynecological . 55
American Laryngological . . . . . . . . . . . . . 55, 767
American Physicians . . . 55
American Surgical Association . . . . . . . . . . 311
Southern Surgical and Gynecological Association . . . . . . . . . 313
Smith: Diseases of Infancy and Childhood . . . . . . . . . . . . 376
Siebert: Physicians' Account Book. 378
Starr: Familiar Forms of Nervous Disease . . . . . . . . . . 306
Diseases of Digestive Organs in Infancy and Childhood. 701
Stemen: Railway Surgery . . . . 191
Treat's Annual and Index . . . . . 769
Ufflemann: Domestic Hygiene of the Child . . . . . . . . . . . 702
Visiting List: The Medical News, for 1891 . . . . . . . 313
The Medical Bulletin . 379
Blakeston's . . . 380
Vulliet et Lutaud's Leçons de Gynecologie Operatoire . . . . . . . 117
Wilder: Health Notes for Students. 115
Witthaus: Medical Students' Manual of Chemistry . . . . . . . . 307
Wood's Medical and Surgical Monographs . . . . . . 250, 377, 631, 768
Woody: Essentials of Medical Chemistry and Urinalysis . . . . . 304

Page.

### Journalistic Notes:

Albany Medical Annals . . . . . . 483
American Microscopic Journal . . . 692
Medical Association Journal. 760
Anales de la Asistencia Publica . . . 503
Annals of Surgery. . . . . . . . . 303
Bacterological World . . . . . . 373
Baltimore Medical and Surgical Record. . . . . . . . . . . . . . 320
Cosmopolitan Magazine . . . . 319, 383
Home-Maker . . . . . . . . . . . 639
International Medical Annual . . . . 448
Journal of Gynecology . . . . . . . 629
King's Journal Directory . . . . . . 320
Ladies' Home Journal . . . . . . . . . . . . . . . . . . 128, 249, 383, 640
Lancet-Clinic . . . . . . . . . . . 760
Literary Digest . . . . . . . . . . 384
Microscope, The . . . . . . . . . 502
Monist, The . . . . . . . . . 249, 382
Neurologisches Centralblatt . . . . . 502
Old Homestead. . . . . . . . . . . 248
Open Court Publishing Co. . . . . . . 384
Pharmacology of the Newer Materia Medica . . . . . . . . . . . . . 190
Review of Insanity and Nervous Diseases. . . . . . . . . . . . . . 303
Revista De Ciencias Medicas De Barcelona . . . . . . . . . . . . . 503
University Medical Magazine . . 248, 369
Vick's Floral Guide . . . . . . . . 511
Western Medical Reporter . . . . . 52

### Editorials:

A Learned Judge . . . . . . . . . 565
American Gynecological Society . . 184
American Medical Association . . . 689
Addresses before . 757
Congress, Inter-Continental 756
Amende Honorable . . . . . . . . . 567
Census of Hallucinations . . . . . 183
Dixie, Doctor . . . . . . . . . . 112
Harderfold Fabric Company's Underwear . . . . . . . . . . . . . . 111
Hay-Fever . . . . . . . . . . . . 107
Heating of Street Cars . . . . . . . 365
Hospital System of Sweden . . . . 180
Hypnotism, Abuse of . . . . . . . 109
Important Medical Legislation . . . 44
In Honor of Dr. Roswell Park . . . 692
Koch's Discovery . . . . . . . . . 299
Koch's Secret Announced . . . . . 435
Medical Corps of the Empire State . 561
Society of the County of Erie . . . . . . . . . . 754
Medical Society of the State of New York . . . . . . . . . . . . . 497

Page.
National Conference of State Medical Examining Boards . . . . . . . 629
New Law Affecting Registration and License of Physicians . . . . . . 46
Niagara University Medical College . 625
New Jersey State Examining Board . 243
One of Those Mischievous Bills . . 623
Protest, A Mild . . . . . . . . . . . 110
Rapid Transit Again . . . . . . . 568
Successful Porro-Cesarean Section . . 49
State Examining and Licensing Board 624

Page.
Two Mischievous Bills . . . . . . . 559
University of Buffalo . . . . . . . . 568
Volume XXX., Alpha . . . . . . . 43
Omega. . . . . . . 754

### New Instruments:

Polyscope . . . . . . . . . . . . 42
Koch's Syringe . . . . . . . . . . 434

